# Cardiac macrophages at the crossroads of inflammation, memory, and repair

**DOI:** 10.1093/immhor/vlag003

**Published:** 2026-02-27

**Authors:** Yiliang Chen

**Affiliations:** Department of Medicine, Medical College of Wisconsin, Milwaukee, WI, United States; Versiti Blood Research Institute, Milwaukee, WI, United States

Macrophages are now recognized as central architects of cardiovascular inflammation and healing. Once viewed primarily as effector phagocytes, they are increasingly understood as dynamic, metabolically adaptable, and epigenetically imprinted cells whose behavior is shaped by spatial cues, past exposures, and their capacity to clear dead cells/cell debris and resolve tissue injury. This special issue of *ImmunoHorizons* brings together three timely reviews that collectively highlight how macrophage diversity, innate immune memory, and efferocytosis converge to influence cardiovascular disease progression and recovery. Together, these articles provide a forward-looking foundation for understanding macrophage biology in the heart and vasculature and identify key challenges for the next phase of research.

Ijaz et al. synthesize recent advances in single-cell and spatial transcriptomics that have reshaped our understanding of macrophage populations within human atherosclerotic plaques.^1^ Their review highlights multiple distinct macrophage states, including inflammatory IL1-β^+^ cells, TREM2^+^ lipid-handling macrophages, PLIN2^+^/TREM1^+^ lipid-stressed cells, HMOX1^+^ macrophages, and LYVE1^+^ resident macrophages, each occupying discrete microanatomic niches within the plaque. Spatial transcriptomics further shows that these subsets localize differentially to the necrotic core, fibrous cap, plaque shoulders, and adventitia, linking cell state directly to microenvironmental cues and disease stage. This work underscores a key emerging theme: **Macrophage phenotype cannot be interpreted apart from spatial context**, and understanding these niches may be essential for developing targeted therapies that stabilize vulnerable plaques.

Hope et al. provide a focused examination of trained immunity—the epigenetic and metabolic reprogramming of innate immune cells that enhances their responsiveness to future stimuli.^2^ Their review reframes the persistent “residual inflammatory risk” observed in cardiovascular patients, demonstrating how endogenous cardiovascular stressors such as hypercholesterolemia, hyperglycemia, hypertension-associated aldosterone, catecholamines released after myocardial infarction, and even weight cycling can induce durable training in monocytes, macrophages, and hematopoietic progenitors. This leads to a long-lasting pool of hyperinflammatory myeloid cells that continue to promote vascular and cardiac inflammation even after traditional risk factors are pharmacologically controlled. The review raises an important clinical question: **Can trained immunity be selectively reversed or modulated without impairing antimicrobial defense?** This remains a significant research frontier with clear translational implications.

**Figure:**
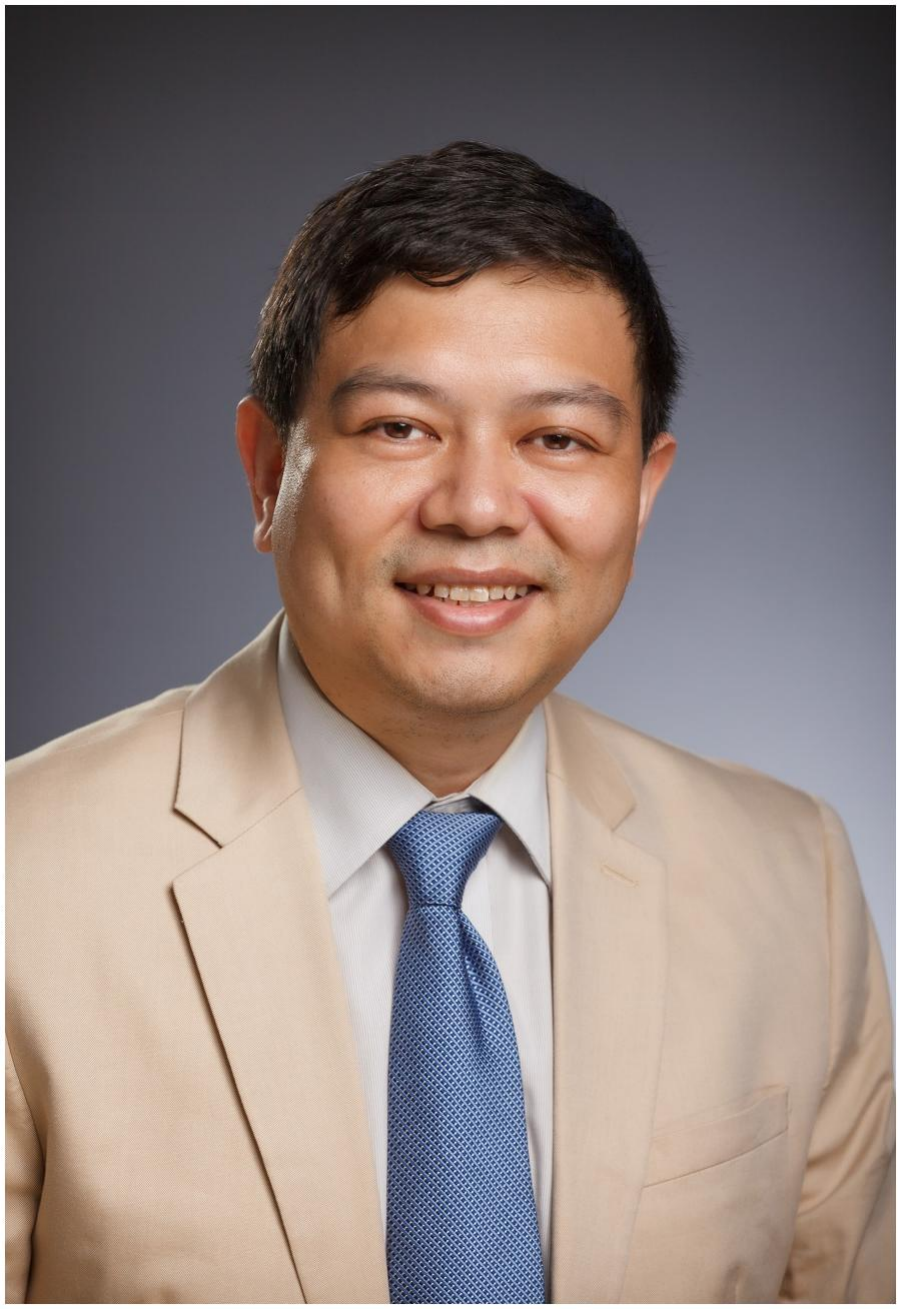
Dr. Yiliang Chen, PhD; Assistant Professor of Medicine, Medical College of Wisconsin, and Associate Investigator at the Versiti Blood Research Institute.

As a type of professional phagocyte, macrophages efficiently clear apoptotic and necrotic cell bodies by a tightly regulated process called efferocytosis. Gupta et al. examine recent literature on macrophage efferocytosis and emphasize its centrality in resolving inflammation and promoting tissue repair.^3^ In atherosclerosis, impaired efferocytosis due to MerTK cleavage, CD47-SIRPα inhibitory signaling, metabolic exhaustion, and age-related decline promotes necrotic-core expansion and plaque instability. Following myocardial infarction, efficient efferocytosis by cardiac macrophages is required to terminate neutrophilic inflammation, support tissue remodeling, and prevent adverse ventricular remodeling. The authors draw attention to emerging therapeutic strategies aimed at restoring efferocytic capacity, including targeting MerTK stability, enhancing pro-resolving mediator pathways, and reprogramming macrophage metabolism. Their review highlights a critical gap: **How can efferocytosis be augmented in advanced or metabolically stressed tissues where macrophages exhibit functional exhaustion?** 

Taken together, this special collection of review articles portrays macrophages as context-sensing, memory-bearing, and reparative immune sentinels whose actions significantly shape cardiovascular outcomes. Several unifying concepts emerge from this collection. First, spatial context is an additional factor in determining macrophage identity and function in vivo. Single-cell spatial technologies reveal that macrophage states are not fixed but arise from microenvironmental cues that vary across plaque regions and cardiac tissue compartments. Second, trained immunity demonstrates that past exposures can significantly shape future responses by macrophages. It provides a previously omitted mechanistic link between systemic cardiovascular risk factors and persistent local inflammation in the heart and vasculature. Third, efficient efferocytosis enables the heart and vasculature to transition from injury toward repair, while its failure drives chronic inflammation and heart disease. Therefore, promoting resolution, rather than suppression, of inflammation may hold the key to solving the problems leading to heart disease.

In conclusion, this special issue highlights a moment of rapid convergence in cardiovascular immunology. By integrating spatial biology, innate immune memory, and cellular mechanisms of resolution, these reviews illuminate how macrophages orchestrate the balance between inflammation and repair in the heart and vasculature. Continued work in these areas holds significant promise for developing next-generation therapies that target macrophage plasticity, restore resolution pathways, and ultimately improve outcomes for patients with cardiovascular disease.
